# Global research on cancer and sleep: A bibliometric and visual analysis of the last two decades

**DOI:** 10.3389/fneur.2023.1139746

**Published:** 2023-03-29

**Authors:** Jiaru Sun, Caihua Wang, Zhaozhao Hui, Wenjin Han, Xiaoqin Wang, Mingxu Wang

**Affiliations:** ^1^Department of Nursing, Xi'an Jiaotong University Health Science Center, Xi'an, China; ^2^School of Public Health, Xi'an Jiaotong University Health Science Center, Xi'an, China

**Keywords:** bibliometrics, cancer, sleep, CiteSpace, visualization analysis

## Abstract

**Objective:**

The study aimed to analyze the research status, hotspots, and frontiers of global research on cancer and sleep through bibliometrics and provide references and guidance for future research.

**Methods:**

The literature regarding cancer and sleep from 2002 to 2022 was searched from the Web of Science Core Collection (WoSCC) database. CiteSpace 5.6.R3 was performed for visualization analysis.

**Results:**

A total of 1,172 publications were identified. The number of publications in the field has gradually increased over the past two decades. The United States had the most prominent contributions. Taipei Medical University and the University of California, San Francisco, and David Gozal were the most prolific institutions and author, respectively. The most published academic journal was *Supportive Care in Cancer*. The research hotspots can be summarized into the symptom cluster intervention for cancer survivors and the association between cancer and melatonin and/or obstructive sleep apnea (OSA). The complex interaction between cancer and sleep disruption and the influencing factors of sleep quality may be the emerging trends of research.

**Conclusion:**

This study systematically analyzed the hotspots and frontiers in the field of cancer and sleep and called for strengthening cooperation among countries, institutions, and authors. In addition, intervention measures for the cancer symptom cluster, the bioavailability of exogenous melatonin, the causal relationship between OSA and cancer, the mechanism of tumor-induced sleep disruption, the dose–response relationship between sleep duration and cancer risk, and the path relationship between sleep quality influencing factors may be the focus of future research.

## 1. Introduction

Cancer is a global public health problem, with a high incidence rate, mortality, and medical expenditure. GLOBOCAN reported that there were an estimated 19.3 million new cases of cancer and nearly 10 million cancer deaths worldwide in 2020, and its burden is expected to increase by 47% (reaching 28.4 million cases) in 2040 (1). In addition, the demand for long-term treatment and the high cost of treatment have posed a substantial economic burden on healthcare systems and survivors (2). As the previous study predicted, total costs of cancer treatment in the US represented a 39% increase over the past 10 years, reaching US $173 billion in 2020 (3). Regarding the out-of-pocket costs for cancer treatment, cancer survivors spent 16 and 42% of their annual income in high-income countries and low- and middle-income countries, respectively (4).

There is a two-way and complex connection between cancer and sleep. On the one hand, sleep disorders may be a risk factor for cancer. Alteration of the circadian rhythm, insomnia, poor sleep quality, and short or long sleep duration were reported to be associated with an increased risk of cancer (5–8). On the other hand, cancer may cause sleep disorders. Extensive evidence demonstrated that cancer survivors develop or aggravate sleep disorders after diagnosis (9, 10). Up to 95% of cancer survivors suffered from sleep disorders during diagnosis, treatment, and after 10 years of survival (10). Among them, insomnia, as the most frequent disorder, had a prevalence rate ranging from 19 to 63% among survivors of various kinds of cancer (11). Individuals suffering from sleep disorders are often accompanied by decreased immune function, pain tolerance, and the ability to fight cancer, which affects the therapeutic effect and also the physical and mental health of survivors (11–14). Notably, numerous studies suggested that both sleep deprivation and prolonged sleep duration were positively associated with the risk of cancer mortality (15–17).

Several pathophysiological mechanisms may have explained the association. First, ghrelin, leptin, glucose, cytokines, certain amino acids, pH, and PCO_2_ can not only control tumor progression through the hypothalamic–pituitary–adrenal axis but also activate the sympathetic nervous system through the brainstem, leading to an alteration of the circadian rhythms (9, 11). Second, melatonin, which is involved in regulating the sleep-wake rhythm, increases the expression of the p53 protein, induces its phosphorylation, and promotes the growth and metastasis of cancer (18). Third, tumors can induce changes in hormone circuits (5-hydroxytryptaminergic, dopaminergic, etc.), leading to sleep disorders (9). Finally, tumors can produce interleukin-1 and tumor necrosis factor alpha to alter sleep rhythms (11).

Considering that the complex relationship between cancer and sleep has not been fully revealed, as well as serious adverse health outcomes, it is significant to review and prospect this research field. Although there have been several reviews on cancer and sleep (10, 11), few studies attempted to analyze the research development in this field through visualization. Visual analysis endows rigorous data with visualization value and delicately depicts the dynamic development and overall structure of a certain field while revealing the research hotspots and frontiers. Based on the bibliometric analysis and visual analysis, this study aimed to summarize the status, hotspots, emerging trends, and dynamic frontiers of global research on cancer and sleep.

## 2. Methods

### 2.1. Data collection procedure

The literature included in the current study was extracted from the Web of Science Core Collection (WOSCC). The search strategy was (sleep) AND ((malignant^*^) OR (oncology) OR (cancer^*^) OR (tumor^*^) OR (neoplasm^*^) OR (carcinoma^*^)). The literature related to cancer and sleep was searched from 2002 to 2022, the document type was selected as “article” and “review”, and the publication language was restricted to “English”. A total of 1,285 publications were retrieved. The manual removal of 113 repetitive and irrelevant papers was done. Finally, a total of 1,172 publications related to cancer and sleep were obtained, and the information on titles, countries, institutions, authors, keywords, and abstracts was extracted. The above screening procedures were performed independently by two researchers.

### 2.2. Analysis tool

CiteSpace software (5.6.R3, 64-bit) was performed for the visualization analysis of the retrieved literature. CiteSpace, invented by Dr. Chaomei Chen (Drexel University, Philadelphia, PA, USA) (19), is an interactive visualization tool combining information visualization methods, data mining algorithms, and bibliometrics. Its visual presentation mode provides scholars with a panoramic perspective on the development of the discipline.

### 2.3. Bibliometric analysis

In this study, we mainly conducted collaboration analysis, keyword cluster analysis, burst keyword analysis, and literature co-citation analysis. The visualization knowledge maps consist of nodes and links. The node type can be set as country, institution, author, keyword, cited references, etc. Its size describes the number of published papers related to this element. The number and thickness of link paths between nodes represent the closeness of the node relationships. The color of the nodes and lines represents different years. Moreover, the centrality reflects the central role and importance of the nodes in the knowledge networks, and the thickness of the purple ring indicates how strong the centrality of the nodes is. We set the parameters of the CiteSpace as follows: (a) timespan from January 2002 to December 2022, year per slice = 1; (b) term source = title/abstract/author keywords/keywords plus; (c) node types = country/institution/author/keyword/reference; and (d) threshold selection criteria = the top 50 items for each time slice. We defaulted to the settings for the other parameters. Modularity Q (Q) > 0.3 indicates a significant cluster structure. In addition, Silhouette (S) > 0.5 means that the clustering result is considered reasonable, and S > 0.7 implies that the clustering result is highly reliable (20).

## 3. Results

### 3.1. Publication years and journals

**Figure 1:**
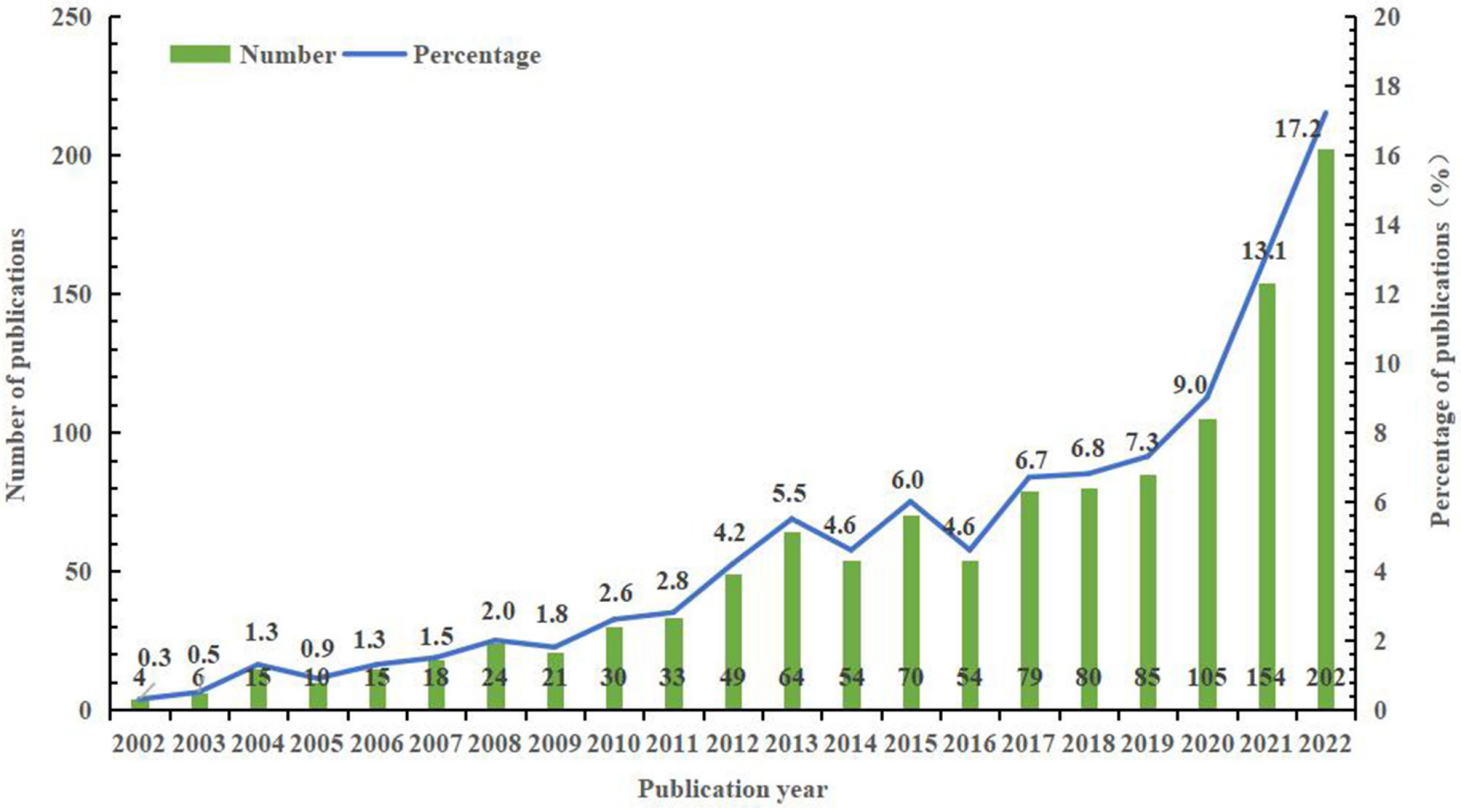
The annual number of publications on sleep and cancer in the WOSCC database from 2002 to 2022.

A total of 1,172 publications were extracted from the WOSCC database from 2002 to 2022, with an overall upward trend in the number of publications over the past 20 years (Figure 1). The lowest output appeared before 2004, with fewer than 10 papers published annually. From 2004 to 2015, the number of publications steadily increased, which demonstrated that the topics of cancer and sleep have gradually attracted the attention of scholars. From 2016 to 2022, the number of published papers has increased rapidly. The number of papers published over the past 5 years accounted for 53.4%, exceeding the cumulative number of papers published between 2002 and 2017. The topic of cancer and sleep is still a hot field.

A total of 1,172 papers related to cancer and sleep were published in 412 academic journals, of which the journal *Supportive Care in Cancer* (*n* = 91, 7.77%) had the highest number of outputs, followed by *Sleep Medicine* (*n* = 44, 3.75%). The top 10 journals with the largest number of publications contributed 28.67% of the total publications, their impact factors (IF) ranged from 1.803 to 11.401, and 50% of the journals were located in the Q1 region (Table 1). Among them, *Sleep Medicine Reviews* had the highest IF (11.401), whereas the average IF of the other journals was approximately 3.954.

**Table 1:** Top 10 most prolific journals in cancer and sleep research.

**No**.	**Journal**	**No. of publications (%)**	**IF^a^**	**JCR^®^ category**
1	Supportive Care in Cancer	91 (7.77)	3.359	Rehabilitation (Q1)
2	Sleep Medicine	44 (3.75)	4.842	Clinical neurology (Q2)
3	Psycho-Oncology	37 (3.16)	3.955	Biomedical (Q2)
4	Journal of Pain and Symptom Management	35 (2.99)	5.576	General and internal (Q1)
5	Cancer Nursing	30 (2.56)	2.760	Nursing (Q1)
6	Sleep	30 (2.56)	6.313	Neurosciences (Q1)
7	Oncology Nursing Forum	23 (1.96)	1.803	Nursing (Q3)
8	Journal of Clinical Sleep Medicine	17 (1.45)	4.324	Clinical neurology (Q2)
9	Sleep Medicine Reviews	15 (1.28)	11.401	Clinical neurology (Q1)
10	Sleep and Breathing	14 (1.20)	2.655	Clinical neurology (Q3)

a Data from the 2022 edition of Journal Citation Reports.

### 3.2. Collaboration analysis

#### 3.2.1. Country/region collaboration analysis

**Figure 2:**
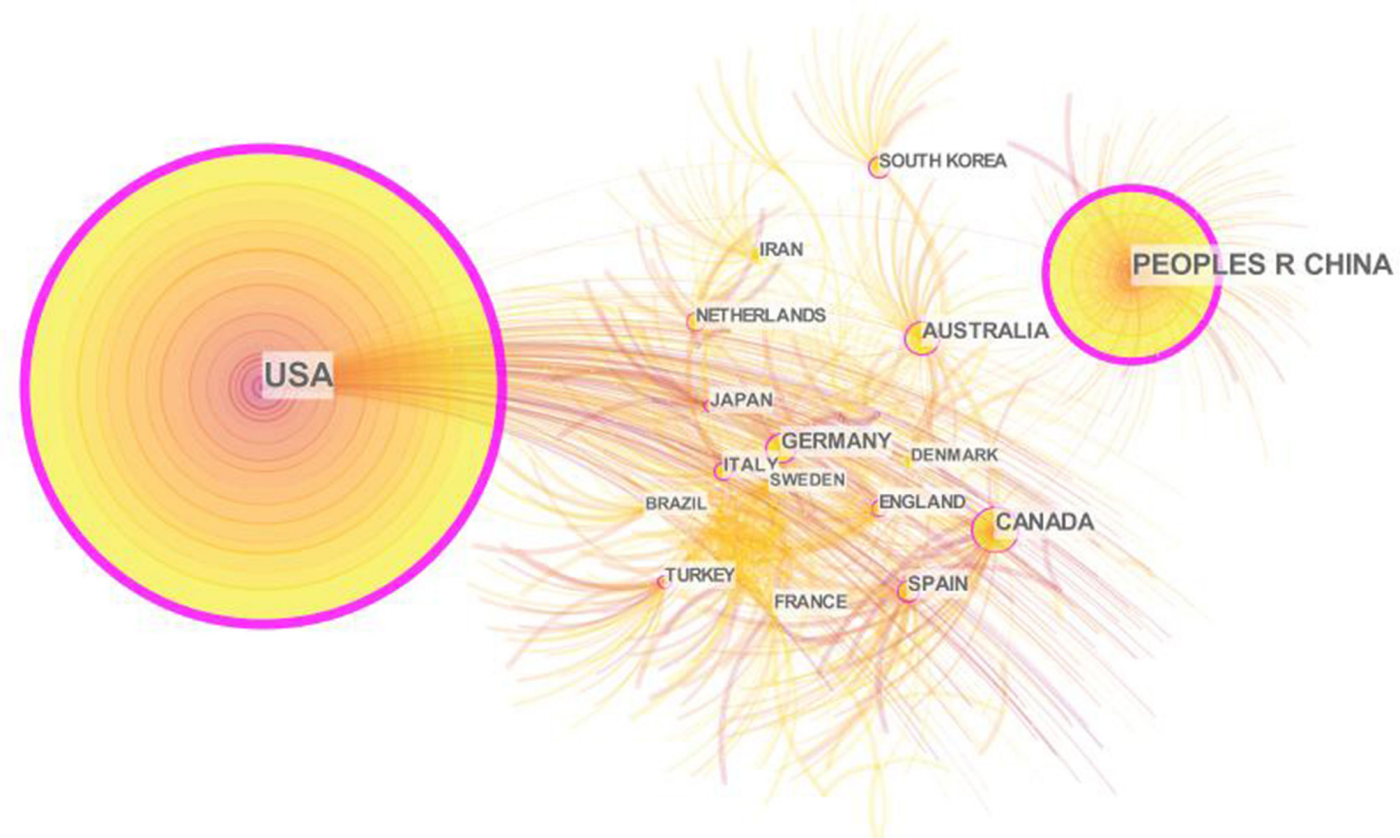
Collaboration network of countries/regions on cancer and sleep research from 2002 to 2022.

This paper set the “country/region” as the node type to analyze the contribution level and cooperation degree of the countries/regions in the field of cancer and sleep (Figure 2). The collaboration network of countries/regions consists of 867 nodes and 1,324 lines, with a network density of 0.0035. The USA was the leading contributor with 518 publications (44.20%), followed by China (*n* = 195, 16.64%) and Canada (*n* = 71, 6.06%). The top three countries published 784 papers on cancer and sleep, accounting for 66.89% of the publications. According to centrality, the United States (0.84) and China (0.62) ranked among the top two and cooperate closely with other countries/regions (Table 2).

**Table 2:** Top 10 countries, institutions, and authors in cancer and sleep research.

**No**.	**Counties/regions**	**Count (%)**	**Centrality**	**Institutions**	**Count (%)**	**Centrality**	**Authors**	**Count (%)**	**Centrality**
1	USA	518 (44.20)	0.88	Taipei Medical University	34 (2.90)	0.07	David Gozal	27 (2.30)	0.03
2	China	195 (16.64)	0.62	University of California—San Francisco	34 (2.90)	0.09	Christine Miaskowski	23 (1.96)	0.00
3	Canada	71 (6.06)	0.15	UT MD Anderson Cancer Center	33 (2.82)	0.05	Ramon Farre	21 (1.79)	0.00
4	Australia	50 (4.27)	0.17	University of Washington	25 (2.13)	0.05	Steven Paul	20 (1.71)	0.00
5	Germany	44 (3.75)	0.17	University of Calif San Diego	24 (2.05)	0.10	Chiachin Lin	19 (1.62)	0.00
6	Spain	40 (3.41)	0.11	Stanford University	24 (2.05)	0.10	Isaac Almendros	19 (1.62)	0.03
7	Netherlands	32 (2.73)	0.11	University of Pennsylvania	22 (1.88)	0.08	Ann M Berger	15 (1.28)	0.01
8	Turkey	31 (2.65)	0.13	Universitat de Barcelona	20 (1.71)	0.08	Kathryn Lee	15 (1.28)	0.00
9	South Korea	30 (2.56)	0.11	National college of Ireland	19 (1.62)	0.05	Claudia West	15 (1.28)	0.00
10	Italy	29 (2.47)	0.12	University of Pittsburgh	19 (1.62)	0.02	Sonia Ancoliisrael	14 (1.19)	0.05

#### 3.2.2. Institution collaboration analysis

**Figure 3:**
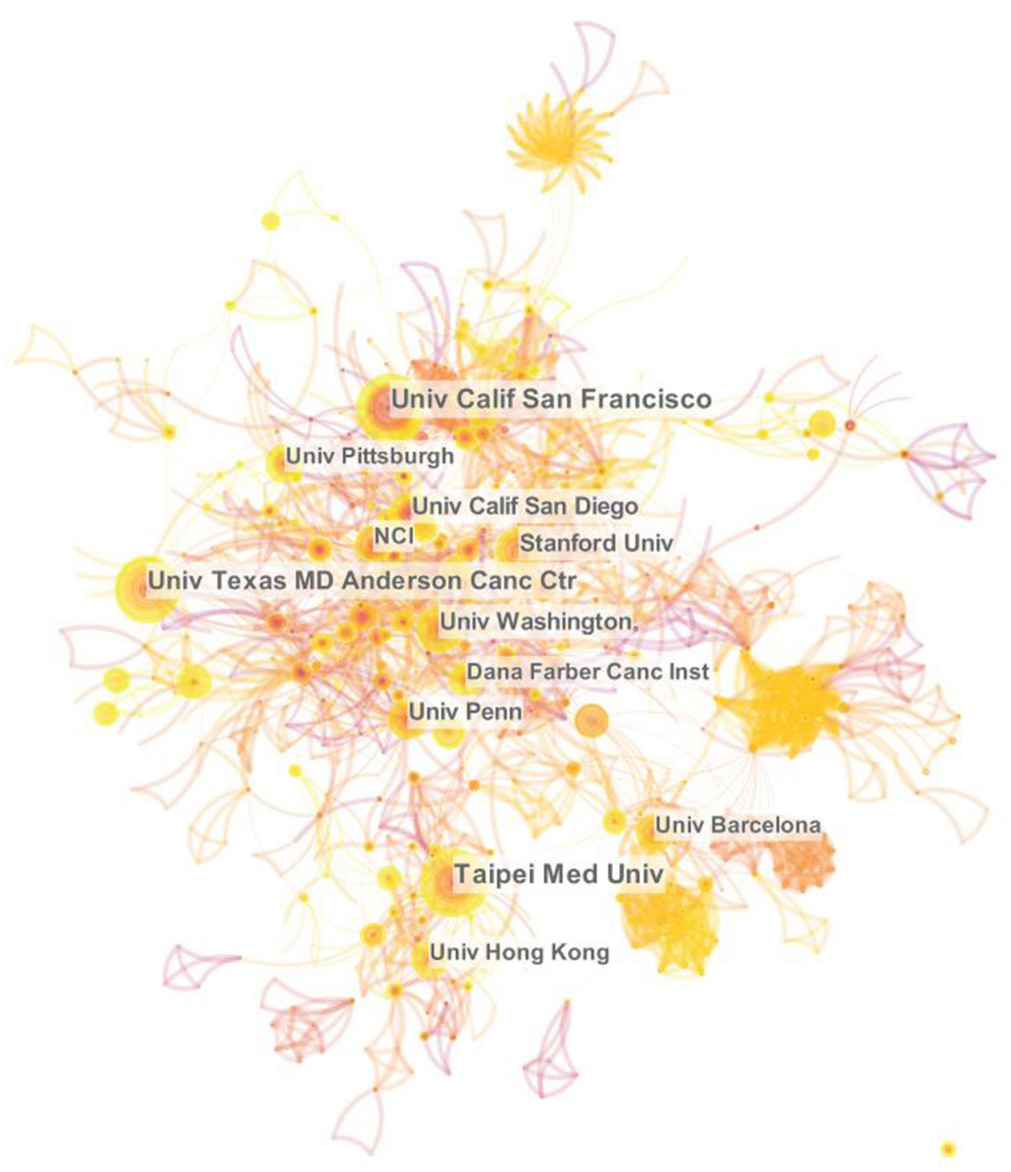
Collaboration network of institutions on cancer and sleep research from 2002 to 2022.

To analyze the cooperative relationships among institutions and identify contributing institutions, this paper generated an institutional network map (Figure 3). The network contains 1,231 nodes and 4,097 links, with a network density of 0.0054. Taipei Medical University and the University of California, San Francisco, published the most papers on this research topic (*n* = 34, 2.90%), followed by UT MD Anderson Cancer Center (*n* = 33, 2.82%). Universities accounted for 90% of the top 10 institutions. In addition, cooperation between institutions was scattered, and only the University of Calif San Diego and Stanford University (0.10) had high centrality (Table 2).

#### 3.2.3. Author collaboration analysis

**Figure 4:**
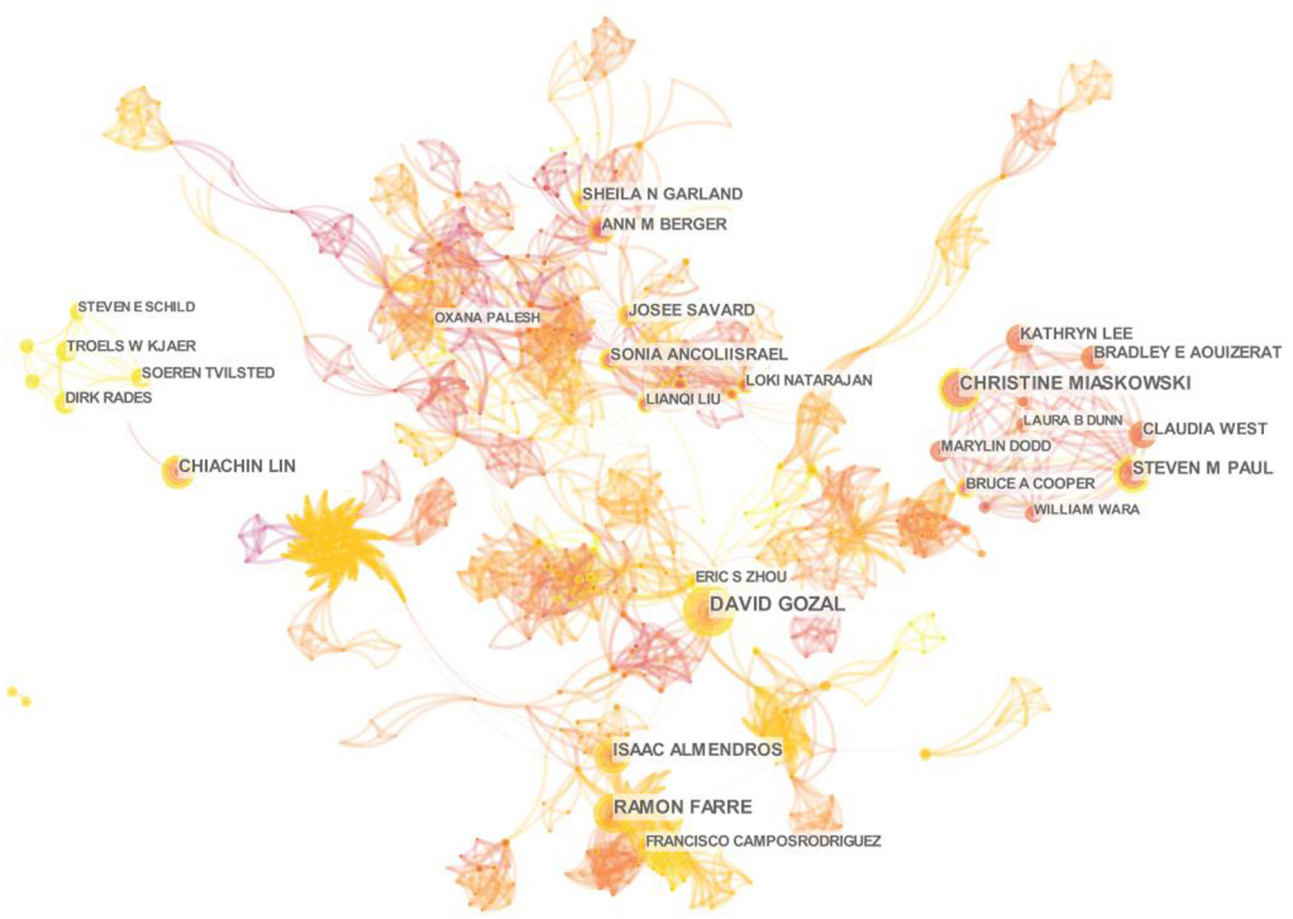
Collaboration network of authors on cancer and sleep research from 2002 to 2022.

To explore the core authors of the research topic and their collaboration, this study drew the author collaboration network (Figure 4). The network contains 3,732 nodes and 12,557 links, with a network density of 0.0018. Although the network density and centrality (both <0.1) indicated less cooperation between scholars, obvious nodes and team relationships can still be observed in the network. Among them, David Gozal contributed the largest number of publications (*n* = 27, 2.30%), followed by Christine Miaskowski (*n* = 23, 1.96%) and Ramon Farre (*n* = 21, 1.79%) (Table 2).

### 3.3. Keyword cluster analysis

**Figure 5:**
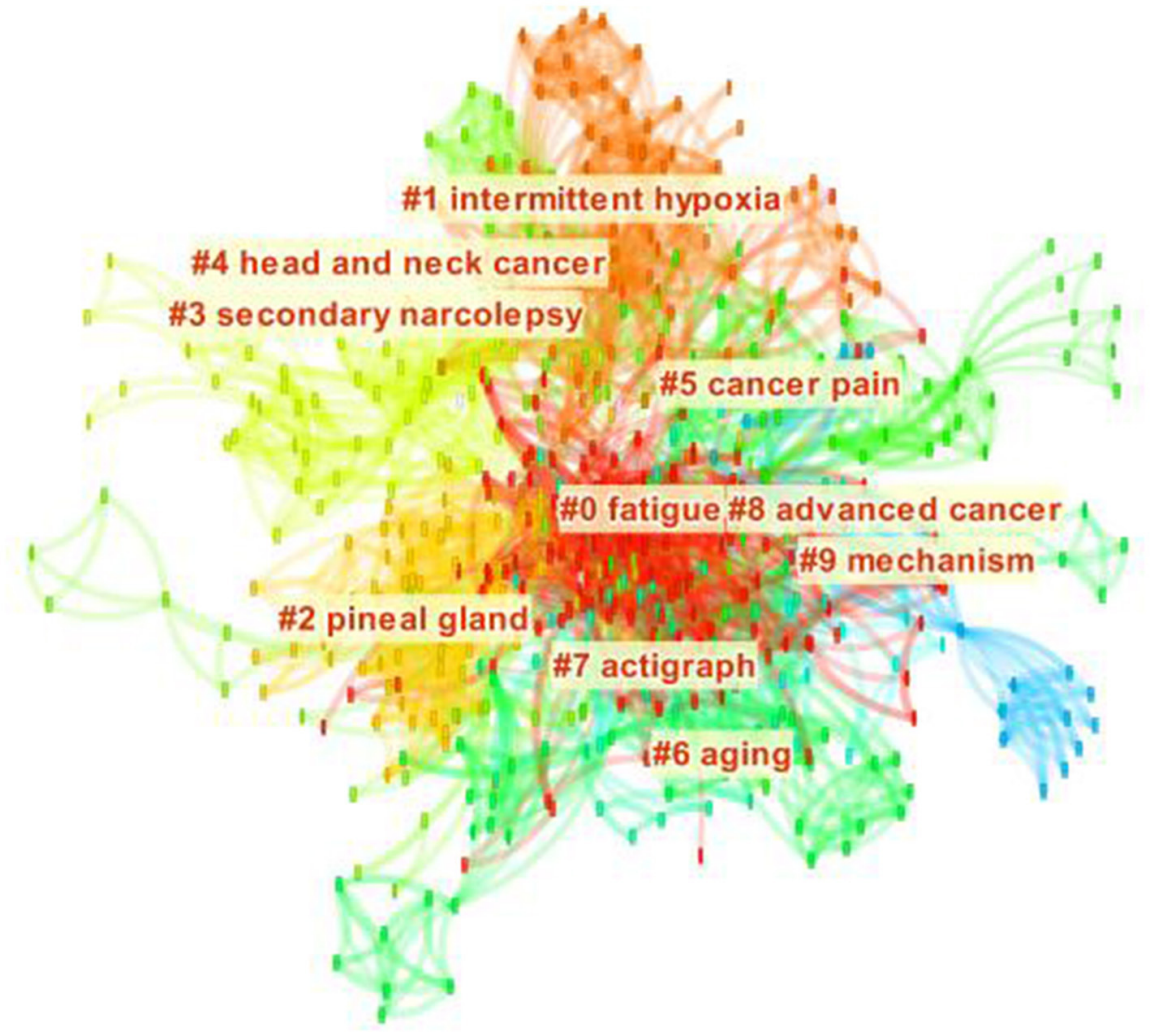
Cluster map of keywords on cancer and sleep research.

The keywords cluster analysis was used to identify the hotspots and emerging trends of cancer and sleep research. The network of cluster keywords consists of 715 nodes and 4,620 lines, with a network density of 0.0181 (Figure 5). The most frequent keywords for cancer and sleep were “breast cancer,” “fatigue,” “insomnia,” “quality of life,” “sleep,” etc. The top five keywords for centrality were “chemotherapy,” “melatonin,” “cancer,” “anxiety,” and “inflammation” (Table 3). The keywords were divided into 10 clusters: “fatigue,” “intermittent hypoxia,” “pineal gland,” “secondary narcolepsy,” “head and neck cancer,” “cancer pain,” “aging,” “actigraph,” “advanced cancer,” and “mechanism”. The clustering was reasonable, with a Q of 0.621, and the S of 0.658.

**Table 3:** Top 10 keywords in terms of count and centrality.

**No**.	**Keywords**	**Count**	**Keywords**	**Centrality**
1	Breast cancer	396	Chemotherapy	0.14
2	Fatigue	362	Melatonin	0.11
3	Insomnia	352	Cancer	0.10
4	Quality of life	335	Anxiety	0.10
5	Sleep	294	Inflammation	0.10
6	Cancer	283	Management	0.08
7	Depression	239	Follow up	0.08
8	Prevalence	209	Colorectal cancer	0.07
9	Women	201	Stress	0.07
10	Disturbance	184	Disease	0.07

### 3.4. Burst keyword analysis

**Figure 6:**
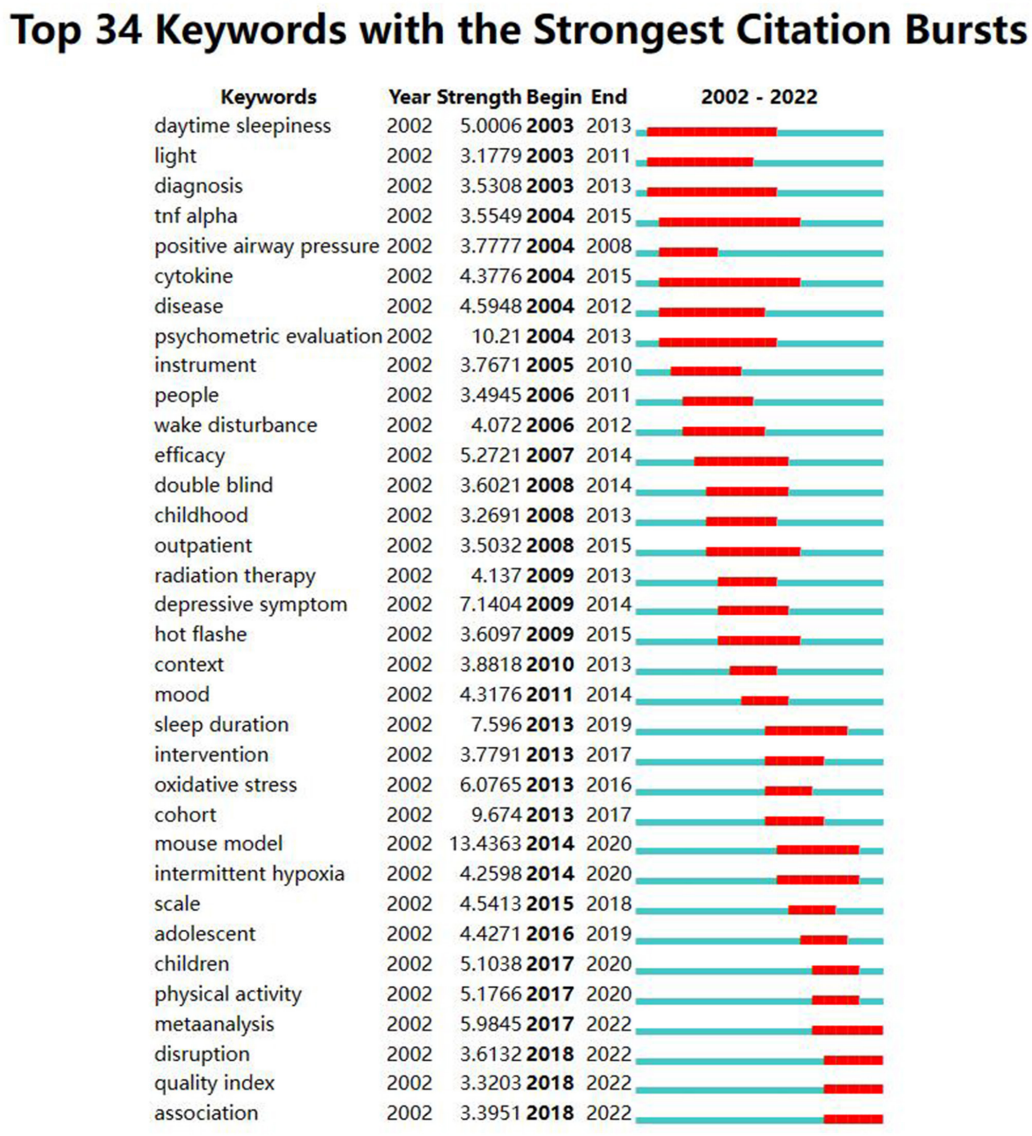
Top 34 keywords with the strongest citation bursts on cancer and sleep research from 2002 to 2022.

The burst keyword analysis was conducted to explore the frontiers of cancer and sleep research. CiteSpace visualized keywords that had significantly increased in number in a short period of time, emphasizing the keywords burst strength and time (Figure 6). This study identified keywords with bursts >4 years in the field of cancer and sleep, i.e., a total of 34, all of which had strength values above 3. Among them, the “mouse model” displayed the highest burst strength, reaching 13.44; “tnf alpha” and “cytokine” had the longest burst duration from 2004 to 2015. The burst time of “disruption,” “quality index,” and “association” has continued today and may become emerging trends in future research.

### 3.5. Co-citation timeline analysis

**Figure 7:**
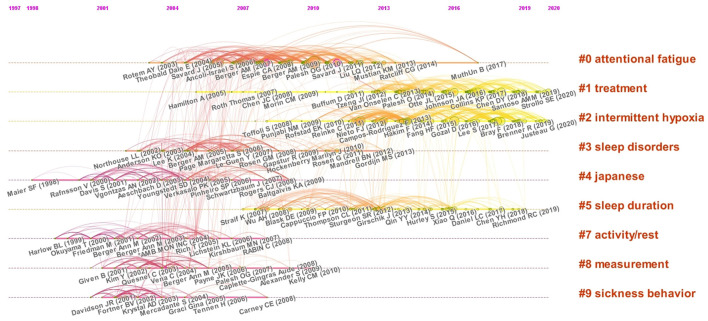
Timeline of co-citation clusters on cancer and sleep research from 2002 to 2022.

The co-citation timeline analysis was performed to summarize the progress and trends in the field of cancer and sleep (Figure 7; Table 4). The co-citation timeline network includes 1,977 nodes and 7,596 links, with a connectivity density of 0.0039. The network was divided into 10 clusters, and the largest cluster was attentional fatigue (#0), followed by treatment (#1), intermittent hypoxia (#2), sleep disorders (#3), Japanese (#4), and sleep duration (#5). The Q was 0.827, and the S was 0.558, meaning the clustering was reasonable. In addition, the number of nodes has gradually increased since 2001, with most clusters focused between 2012 and 2020. The highest concentration of nodes with citation bursts was cluster #0 attentional fatigue, followed by cluster #1 treatment and cluster #7 intermittent hypoxia.

**Table 4:** The top 10 cited references in cancer and sleep research.

**No**.	**Count**	**Centrality**	**Title**	**Author**	**Journal (IF^a^, JCR^®^ category)**	**Published year**
1	47	0.03	Prevalence, demographics, and psychological associations of sleep disruption in patients with cancer: University of Rochester Cancer Center-Community Clinical Oncology Program.	Palesh OG, et al.	J Clin Oncol (50.717, Q1)	2010
2	41	0.00	Global cancer statistics 2018: GLOBOCAN estimates of incidence and mortality worldwide for 36 cancers in 185 countries.	Bray F, et al.	CA-Cancer J Clin (286.130, Q1)	2018
3	38	0.03	Sleep-disordered breathing and cancer mortality: results from the Wisconsin Sleep Cohort Study.	Nieto FJ, et al.	Am J Resp Crit Care (30.528, Q1)	2012
4	37	0.01	Obstructive sleep apnea and cancer: Epidemiologic links and theoretical biological constructs.	Gozal D, et al.	Sleep Med Rev (11.401, Q1)	2016
5	35	0.00	Reply: Obstructive sleep apnea and cancer: is it time to study organ-specific cancers?	Campos-rodriguez F, et al.	Am J Resp Crit Care (30.528, Q1)	2013
6	31	0.01	Intermittent hypoxia enhances cancer progression in a mouse model of sleep apnoea.	Almendros I, et al.	Eur Respir J (33.795, Q1)	2012
7	30	0.01	Measurements and status of sleep quality in patients with cancers.	Chen DY, et al.	Support Care Cancer (3.359, Q1)	2018
8	30	0.02	A systematic review and meta-analysis of randomized controlled trials of cognitive behavior therapy for insomnia (CBT-I) in cancer survivors.	Johnson JA, et al.	Sleep Med Rev (10.602, Q1)	2016
9	29	0.00	Intermittent hypoxia increases melanoma metastasis to the lung in a mouse model of sleep apnea.	Almendros I, et al.	Resp Physiol Neurobi (2.821, Q3)	2013
10	28	0.01	Sleep apnea and subsequent cancer incidence.	Sillah A, et al.	Cancer Cause Control (2.532, Q3)	2018

^a^Data from the 2022 edition of Journal Citation Reports.

This paper listed 10 representative references related to cancer and sleep (Table 4). The top 10 references were published from 2010 to 2018. About 80% of the top 10 references were published in top journals in Q1, and the references with IF higher than 30 accounted for 60%. The top three references were papers with the largest number of citations (*n* = 48), the highest IF (186.130), and the highest centrality (0.03), respectively. Among them, the study by Palesh OG, et al. was a prospective study exploring the prevalence of insomnia and related factors in patients with cancer receiving chemotherapy. The study by Bray F et al. calculated the incidence rate and mortality of 36 cancer types in 185 countries. The cohort study of Nieto FJ et al. analyzed the association between sleep-disordered breathing and cancer mortality. The remaining seven reference types include review or meta-analysis (*n* = 3), comment (*n* = 1), cohort study (*n* = 1), and basic studies based on mouse models (*n* = 2).

## 4. Discussion

To the best of our knowledge, this is the first study to visualize the research status, hotspots, and frontiers of global research on cancer and sleep through bibliometrics. The number of publications in the field has gradually increased over the past two decades and has shown a continuously rising trend. The studies were mainly published in journals related to cancer care or sleep. The United States and China, as well as universities, have contributed the most to this field, with the highest number of publications and influences. David Gozal has published the most papers. Although there was a certain team cooperation network, the cooperation between the authors was still scattered and lacked stability. In addition, the research hotspots in this field can be summarized into the symptom cluster intervention for cancer survivors and the association between cancer and melatonin produced by the pineal gland and/or intermittent hypoxia (that is obstructive sleep apnea, OSA). The Complex interaction between cancer and sleep disruption and the influencing factors of sleep quality may be the emerging trends of research.

The United States has occupied a dominance in the field of cancer and sleep, reflecting the superlative centrality and the far leading number of publications. The second was China and Canada, which is consistent with the previous bibliometric studies on the sleep pattern of college students (21). Among the top 10 productive countries, only China and Turkey are developing countries. Research showed that the standardized incidence of cancer in transitioning economies was 200–300% higher than in transitioning economies (22, 23). The incidence rate and mortality of cancer in China and Turkey are getting shifted to developed countries (24, 25). As a large country with a population of 1.4 billion, the increase in incidence rate or mortality in China will cause incalculable serious consequences. Therefore, we suggest that Chinese and Turkish scholars, especially the former, to conduct more cooperative research with other developed countries. Then, of the top 10 institutions, 70% came from the USA and 90% were universities. The cooperative network maps of both the institutions and the authors were relatively scattered. Accumulating evidence suggested that more inter-institutional communication and collaboration between authors may be related to higher research productivity and research quality (26, 27). Consequently, it is necessary to expand the cooperation network between institutions and authors, especially with American universities.

Keyword clustering summarized the core and hotspots of the research field. Head and neck cancer, the elderly, and advanced cancer were the hot populations in the field of cancer and sleep. There were three main research directions in the field, namely, basic research, influencing factors, and intervention measures, but few studies are based on the epidemiology of sleep disorders in all types of cancer survivors. It is worth noting that symptom cluster interventions were the most popular topic. A study demonstrated that more than 40% of cancer survivors were accompanied by the symptom cluster of pain, fatigue, and sleep disorders, and may persist throughout the survival period (28, 29). Currently, interventions for cancer-related pain, fatigue, and sleep disorders can be summarized in four aspects: exercise interventions, cognitive behavior therapy (CBT), stimulation therapies, relaxation interventions, and pharmacologic therapy. Some interventions can only work in one or two symptoms (30, 31). The other interventions, such as 3, 6, and 9 months of resistance and aerobic exercise combined training, CBT, slow-stroke back massage three times a week for 4 weeks, and music therapy for 5 days, were reported to improve the whole symptom cluster (32–35). However, the time, intensity, cycle, and feasibility of the intervention need to be further verified. Researchers should conduct high-quality randomized controlled studies in different cancer types to provide effective interventions to alleviate the cancer-symptom cluster. Another popular topic was the relationship between melatonin produced by the pineal gland and cancer. Melatonin has recently emerged as an important inhibitor in cancer occurrence and development, which has been reported in various fields, including breast cancer, colorectal cancer, lung cancer, prostate cancer, ovarian cancer, and oral cancer (36–41). Based on this, many scholars have shifted their attention to the treatment of cancer with melatonin. Several studies indicated that melatonin has benefits in cancer treatment, such as enhancing the therapeutic effect of anti-cancer drugs and improving cognitive flexibility, attention, sleep quality, insomnia, and quality of life in cancer survivors (40, 42, 43). However, the bioavailability, pharmacokinetic properties, and interindividual differences of exogenous melatonin deserve further understanding (40). The third research hotspot was the association between OSA and cancer. Although experimental studies in animal or cellular models provided strong clues to OSA causes of cancer (44, 45), the epidemiological evidence remains insufficient and inconsistent (46–50). In future, longitudinal studies should be conducted to verify the causal relationship between OSA and cancer, taking into account age, cancer type, obesity, and other factors (50, 51).

Burst keywords and their changing trends can reflect the development process and frontiers of research to a certain extent. The development process of research can be divided into two stages. From 2002 to 2012, the research was at the initial stage, mainly focusing on the status of sleep disorders in cancer survivors. Since 2013, the field of cancer and sleep has developed rapidly. Scholars paid more attention to the influencing factors and interventions of cancer-related sleep disorders and attempted to elaborate on the mechanisms underlying them through basic research such as mouse models. The research objects were also more refined, ranging from people to outpatients, adolescents, and children. More importantly, the burst strength of keywords including “meta-analysis,” “disruption,” “quality index,” and “association” was stronger than three, and the burst time has continued today and may remain ongoing in the future. We sum up the two other research trends: first, the complex interaction between cancer and sleep disruption. The causal relationship between them was unclear similar to the “chicken or the egg” problem. Studies have shown that tumor metabolism and its cytokines can lead to sleep interruption (9). On the contrary, sleep interruption can promote the occurrence and development of cancer through anti-tumor immunity, inflammatory response, metabolism, and sympathetic nervous system (52). The mechanism of the former needs to be further refined and supplemented, while the relevant epidemiological evidence of the latter is inconsistent (53–56). In future, well-designed prospective studies are needed to explore the dose–response relationship between sleep duration and cancer risk, as well as the performance in different types of cancer. Second, the influencing factors of sleep quality can be summarized into five aspects: demographic factors (advanced age, female, race, lower education level, etc.), disease and treatment factors (distant metastatic cancer, usage of opioids, radiotherapy, chemotherapy, etc.), symptom cluster (fatigue, pain, etc.), psychologic factors (anxiety, depressive symptoms, etc.), and other factors (OSA, BMI, restless legs syndrome, ward environment, etc.). At present, the research results of some influencing factors are not uniform, and the path between influencing factors and sleep quality also needs to be further explored through mediation research (38, 57–61).

There are some limitations to the current study. First, we only searched the publications from the WoSCC database; however, cancer and sleep related literature from other large databases such as PubMed or Embase were not included. Due to the different properties of different databases (62), it may be inappropriate to merge papers from multiple databases. Moreover, the WoSCC database is the most representative and cutting-edge authoritative database, which contains the world's most prestigious high-impact scholarly journals. Second, limited by the CiteSpace software, this study only included articles published in English in the last two decades, which may not be comprehensive. Third, there were no clear criteria to review the quality of over 100 publications, so articles of low quality cannot be excluded from further analysis.

## 5. Conclusion

This study provided a systematic visualization analysis of the field of cancer and sleep. Strengthening cooperation among countries, institutions, and authors will help improve the quality and output of research. Moreover, the current research hotspots are the symptom cluster intervention for cancer survivors and the association between cancer and melatonin and/or OSA. The complex interaction between cancer and sleep disruption and the influencing factors of sleep quality are considered to be emerging trends, which deserve our further attention. This study promoted the transformation of research results into clinical practice and provided a reference for scholars to break through research boundaries and determine research directions.

## Author contributions

MW and XW conceived the study design. CW contributed to data acquisition. JS performed the data analysis and wrote the manuscript. ZH and WH participated in the revising of the manuscript. All authors have read and agreed to the published version of the manuscript.
